# Right-Sightedness and Left-Sightedness

**Published:** 1894-07

**Authors:** M. De Camarasa


### SELECTIONS.

### Right-Sightedness and Left-Sightedness.

BY M. DE CAMARASA.

Are you right-sighted or left-sighted? Nowadays, good shots generally shoot with both eyes open. How are they able to aim, —that is, to bring the object, the two sights of the gun, and the eyes in line? It is impossible to bring more than one eye into the line of sight. Nevertheless, sportsmen assure you that they sight with both eyes, and in fact, at the moment of firing they have both eyes open.

To test the matter, take a piece of card or stiff paper, and make a hole through it with a slender pencil, and, holding it at a little distance before your eyes, look through it, with both eyes open, at a spot on another card lying before you on the table. When you sight this spot, close first one eye, then open it and close the other, and you will find that one eye only is in line with the hole and the dot. It is the same with the sportsman who shoots with both eyes open, one eye only is brought into play. The experiment may be varied by pointing with the finger at a small object several yards distant, when, on closing each eye in turn, it will be found that only one of them is in the line of sight.

Many sportsmen who shoot with both eyes open are excellent shots, and many who formerly shot with one eye closed have changed their method. They are convinced that shooting with both eyes open has real advantages. The object can be seen better, and the distance calculated better, and, at the moment of pressing the trigger, the muscular effort necessary to the closing of one eye is avoided. Many persons, indeed, are unable to close each eye in turn while keeping the other open. Moreover, people are right-sighted or left-sighted just as they are right-handed or left-handed; that is to say, one eye is more efficient than the other. A person whose right eye is deficient must modify a little the position of the head or of the arms, in order to bring the left eye into line. But there are guns made especially for sportsmen in whom the sight of the right eye is defective, the stocks being so bent that the left eye can be used for sight without change of the normal position. The stock and barrels in fact are aligned on different planes, the distance between which is equal to the distance between the two eyes.

When a gun-maker is making a gun for a person who shoots with both eyes open, it is, therefore, important to know whether the marksman is right-sighted or left-sighted. Of course a left-sighted person may learn to shoot from the left shoulder, but for a right-handed man this requires an apprenticeship. As a matter of fact, most people are right-sighted, but the exceptions are sufficiently numerous to render it prudent to determine the point before having a gun made to order. It is of course possible to bring the other eye into the line of sight, but the stronger eye falls naturally into it.

But the prime object of this article is not to discuss the advantages and inconveniencies of shooting with one eye or with both eyes open, whether with weapons of the chase or with weapons of war. I am led to the subject simply because I was assured by an old soldier that he had spent many days under arrest because he was not able to close the left eye when taking aim. The case is assuredly not an isolated one; and I ask myself, would it not be more rational to instruct military marksmen to shoot, as so many civilians do, with both eyes open, a method which does away at once with needless efforts, grimaces and possible punishment?

Apart from this, marksmen are not the only persons who, having to work with one eye at a time, work with both eyes open, even for the most delicate work; watchmakers, microscopists and others do the same successfully, and thereby effect an economy of effort.

In the course of my experience, I have met a great many people who have never thought of whether they are right sighted or left-sighted. I believe, however, it may be asserted without fear of error, (1) that it is possible to utilize one eye only while both are open ; (2) that there are right-sighted and left-sighted persons; (3) that a person is ordinarily ignorant of whether he is right-sighted or left-sighted until he has made it a matter of experiment; and (4) that the eye to which a person devotes the attention and the will—in other words, the eye with which he regards an object—is the one with which he sees it.

The last fact is sufficiently demonstrated by the experience of microscopists. It may be also verified by taking two paper tubes, putting one to each eye, opening them at a considerable angle, so as to allow the eyes to fall on two pages of reading-matter at some distance apart. By an effort of will one can read with either eye, but while one eye is occupied the other rests.—*La Nature, Paris, April* 14.

